# Express assessment of neurotoxicity of particles of planetary and interstellar dust

**DOI:** 10.1038/s41526-019-0062-7

**Published:** 2019-02-04

**Authors:** Tatiana Borisova

**Affiliations:** Department of Neurochemistry, Palladin Institute of Biochemistry, National Academy of Sciences of Ukraine, 9 Leontovicha Street, Kiev, 01030 Ukraine

## Abstract

Establishment of high-quality, consistent on-board assessment of the neurotoxicity of planetary, and interstellar dust particles will be required to predict their potential threat to human health during long-term space missions. This Perspective article proposes an approach for the rapid assessment of potential neurotoxicity of micro-sized and nano-sized dust particles based on experimental results with other neurotoxic particles. Capacity of particles to affect membrane potential, integrity of nerve terminals, and consequently key synaptic transmission characteristics can be assessed using a planar lipid bilayer technique by monitoring artificial membrane conductivity in the presence of particles. Preliminary neurotoxicity data of nanoparticles, including lunar and Martian dust simulants, obtained using a planar lipid bilayer technique, is compared with that acquired using more-established methodological approaches. Under space flight conditions, neurotoxicity assessments of particulate matter could be rapidly and reproducibly performed using a planar lipid bilayer technique, which does not require biological material.

## Introduction

Particles of planetary and interstellar dust might affect human health during long-term human space missions, so their toxicity risk assessment is required for human planetary exploration. Throughout several Apollo missions, lunar dust particles were shown to adhere to space suits, thereafter getting into spacecraft and having a direct contact with the astronauts.^1^ These particles caused irritation of eyes, the respiratory system, and skin.^1^ Additionally, these dust simulants damaged alveolar macrophages in vitro and elicited an increase in cytotoxicity.^2^ An acute inflammatory response led to a chronic inflammatory lesion during prolonged presence of lunar and Martian dust simulants in the lungs of mice.^3^ Expression of inducible nitric oxide synthase in a murine macrophage cell line was also enhanced by lunar dust simulant.^4^ It was shown evaluating the respiratory system of mice that lunar dust simulant was more toxic than titanium dioxide particles, while the acute effects of Martian dust simulant were comparable with those of quartz dust.^3^ Assessments of biochemical and cellular markers of toxicity in lavage fluid and histopathology of lungs and lymph nodes showed that the highest Non-Observable-Adverse-Effect Level (NOAEL) was 6.8 mg/m^3^ for rats exposed during 4 weeks to lunar soil aerosols collected during the Apollo 14 mission; an estimated human NOAEL was 2.3 mg/m^3^.^5^ Other study on assessment of the pulmonary toxicity of airborne lunar dust in rats exposed by nose-only inhalation showed safe exposure estimate values ranging from 0.6 to 0.9 mg/m^3^, when benchmark dose was extrapolated to humans.^6^ Notable, current air quality standards of the European Commission for air pollution particulate matter (PM) are 25 μg/m^3^ for PM with an aerodynamic diameter below 2.5 μm (PM_2.5_) and 40–50 μg/m^3^ for PM_10_.^7^

Toxicity assessment of engineered particles and environmentally derived micro-sized (PM_0.1–2.5_) and nano-sized (PM_<0.1_) PM is a focus of research interest, particularly as it pertains to effects on the central nervous system (CNS). Accumulating evidences suggest that inhaled outdoor air pollutants may have a considerable impact on health and disease of the CNS.^8^ More recently, environmental exposure to PM_2.5_ has been associated with autism, children’s attention deficit/hyperactivity disorders, and neurodegenerative diseases, including dementia in adults—although evidence of causation has not been established for these disorders.^9–12^

In 2016, exogenous magnetite nanoparticles were found present in the human brain, likely via uptake through the olfactory bulb.^13^ Nanoparticles can accumulate in the nasal regions, where they are then transported along sensory axons of the olfactory nerve directly to the CNS.^14,15^ Titanium dioxide nanoparticles intranasally instilled to mice can be translocated to the brain and cause potential lesions, with the hippocampus being the main target.^16^ Further, a mouse study suggested that nanoparticles could be transferred from mother to child. Titanium dioxide nanoparticles administrated subcutaneously to pregnant mice were registered in the brains of the offspring, had a severe negative effect on fetal brain development and a risk of various nervous system disorders.^17^

While the ability of micro-sized and nano-sized particles to circumvent the blood–brain barrier is encouraging for nanomedicine, this route presents problems regarding potential neurotoxic effects of planetary and interstellar dust for the safety of human space flight. A rapid and accurate system for toxicity assessment of these dust particles under space flight conditions would be useful to address this concern. Dust particle toxicity should also be analyzed in situ as different planetary regions have their own unique environmental factors (for example, ultraviolet and ionizing radiation) which, in turn, can significantly influence particle properties.^18–20^

The PM sensors currently used for Earth environmental monitoring detect PM_1_ (starting to be monitored), PM_2.5_, and PM_10_, with no discrimination regarding chemical composition.^7^ Lead, cadmium, nickel, arsenic, and polycyclic aromatic hydrocarbons are monitored separately.^7^ These monitoring techniques can be useful to collect information during space exploration, but they are not enough to predict the potential toxicity of planetary and interstellar dust. Indeed, detailed investigation of toxicity requires laboratory testing, which can be performed using highly sophisticated equipment (mass spectrometry^21^, laser correlation spectroscopy^22^, electron^22^, and confocal laser scanning microscopy^23^) and a variety of different cell culture types, tissue preparations, and animals. However, bulky equipment and biological material are not practical for on-board measurements during space flight.

Therefore, the aims of this Perspective article are to first outline the potential for analysis and comparison of changes in the membrane potential of nerve terminals induced by different types of micro-sized and nano-sized particles and consequent alterations of key synaptic transmission characteristics. In addition, this Perspective describes an approach for rapid assessment of potential neurotoxicity of micro-sized and nano-sized dust particles for use in space flight.

## Neurotoxicity of different types of particles

Characterization of the neurotoxicity of engineered and environmentally derived micro-sized and nano-sized particles is incredibly useful for designing an adequate approach that could assess the potential toxicity of planetary and interstellar dust. Experiments concerning neurotoxicity risk assessment have been performed at the neurochemical level of nervous system organization and involved an analysis of neurotransmitter transport, functioning of ion channels, and other parameters (according to Guidelines for Neurotoxicity Risk Assessment of US Environmental Protection Agency, 1998, based on paragraph 3. Hazard Characterization: 3.1.2. Animal Studies; 3.1.2.3. Neurochemical Endpoints of Neurotoxicity; 3.1.3.4. In Vitro Data in Neurotoxicology).^22–29^ Nerve terminals isolated from rat brain (synaptosomes) have been used as models when exploring the relationship between the structure of synaptic proteins, their biochemical properties, and physiological roles.^30^ The effects of different types of nanoparticles on the transport characteristics of radiolabelled glutamate and γ-aminobutyric acid (GABA), which are key excitatory and inhibitory neurotransmitters in the CNS, respectively, were examined using synaptosomes.^22–29^ Studies have revealed three types of functional changes in synaptosomes in response to application of different nanoparticles: (1) decreased exocytotic release and Na^+^-dependent neurotransmitter transporter-mediated uptake L-[^14^C]glutamate^31^ and [^3^H]GABA, (2) elevated tonic and transporter-mediated release, and ambient levels^32^ of these neurotransmitters, (3) attenuation of acidification of synaptic vesicles and resting membrane potential.^22–29^ Different particles elicit varying combinations of these effects in synaptosomes. For instance, the levels of L-[^14^C]glutamate binding to synaptosomes in low Na^+^ concentration media and at low temperature increased in the presence of simulated lunar soil, whereas simulated Martian soil caused significantly less changes under the same conditions (both simulated soils are composed of natural volcanic ash). This suggests that lunar dust interacts with the synaptosomal plasma membrane to increase non-specific L-[^14^C]glutamate binding.^22^ Using the same methodological approaches, the neurotoxic and neuromodulatory properties of carbon nanodots synthesized from β-alanine,^23^ thiourea^24^, and detonation nanodiamonds^25^ were predicted. The mixture of simulated Martian soil particles and carbon nanoparticles, potential components of native Martian dust, impaired uptake and release of excitatory and inhibitory neurotransmitters, and so had a harmful effect on synaptic neurotransmission. This effect was determined by its carbon content, but not inorganic components.^26^ Also, the study demonstrated the ability of NaYF_4_:Eu^3+^nanocrystals^27^ and maghemite nanoparticles γ-Fe_2_O_3_^28,29^ to change neurotransmitter transport characteristics in synaptosomes, namely uptake and release of excitatory and inhibitory neurotransmitters. Acquired experimental data define a representative parameter that is indicative of acute neurotoxicity causing by nanoparticles (see below).

## Nerve terminal membrane potential

**Fig. 1:**
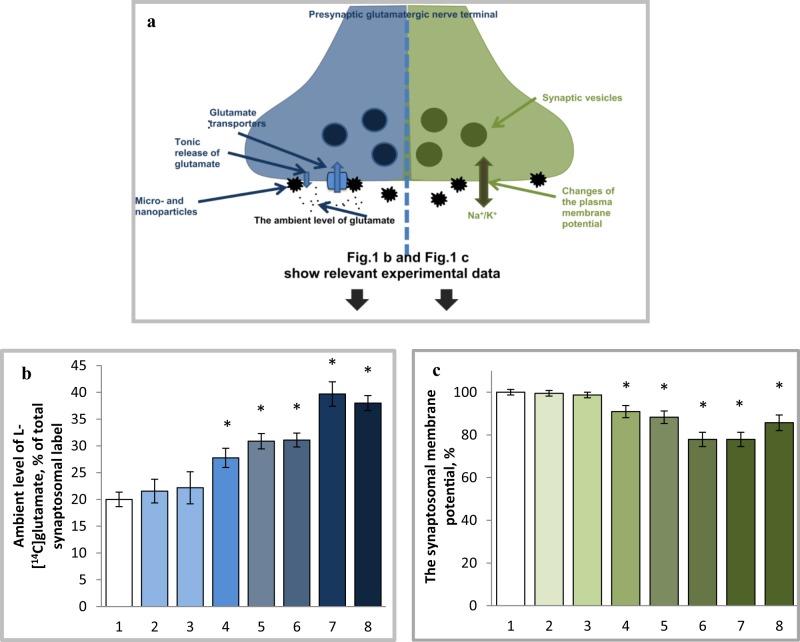
Correspondence of an increase in the ambient level of L-[^14^C]glutamate (**a**, **b**) reflecting the neurotoxic potential of nanoparticles and a decrease in the membrane potential registered using fluorescent dye rhodamine 6 G (**a**, **c**) in the preparations of nerve terminals without (**b**, **c**, columns # 1) and in the presence of micro- and nano-sized particles: simulated Martian soil, 2.0 mg/ml (**b**, **c**, columns # 2);^22^ Lunar soil simulant, 2.0 mg/ml (**b**, **c**, columns # 3);^22^ nanocrystals NaYF_4_:Eu^3+^-PEG, 7.5 mg/ml (**b**); 3.5 mg/ml (**c**) (columns # 4);^27^ nanocrystals NaYF_4_:Eu-^3+^OH, 3.5 mg/ml (**b**); 1.5 mg/ml (**c**) (columns # 5);^27^ maghemite nanoparticles, 0.75 mg/ml (**b**, **c**, columns # 6);^28^ nanodiamonds, 1.0 mg/ml (**b**, **c**, columns # 7);^25^ carbon nanodots synthesized from thiourea, 1.0 mg/ml (**b**); 0.5 mg/ml (**c**) (columns # 8).^24^ Data are mean ± SEM of six independent experiments. *p < 0.05, as compared to the control; Student *t-*test

The plasma membrane of nerve cells is directly involved in nerve signal transmission and simultaneously performs a classical barrier function, which is interrupted during contact with dust particles.^33^ Changes of key neurotransmitter transport parameters (exocytotic, tonic, and Na^+^-dependent neurotransmitter transporter-mediated release of L-[^14^C]glutamate and [^3^H]GABA; uptake and the ambient level of these neurotransmitters) correlated with alterations of the membrane potential (measured using fluorescent potential-sensitive dye) during exposure of synaptosomes to carbon nanodots,^24^ detonation nanodiamonds,^25^ NaYF_4_:Eu^3+^nanocrystals,^27^ and γ-Fe_2_O_3_ nanoparticles.^28,29^ Interestingly, simulated lunar and Martian dusts did not affect the membrane potential and were inert regarding neurotransmitter transport modulation.^22^ The increase in non-specific glutamate binding induced by simulated lunar dust did not correlate with any change in membrane potential.^22^ The mixture of simulated Martian soil particles and carbon nanoparticles depolarized the plasma membrane^23–26^ and consequently modulated neurotransmitter transport.^26^ Thus, nanoparticle-induced changes in key synaptic transmission characteristics and the neurotoxic features of different types of particles examined in our experiments corresponded to their ability to change the plasma membrane potential of synaptosomes (Fig. 1)(Table 1). These experimental data suggest that the plasma membrane potential of synaptosomes can be used as a representative parameter to adequately reflect acute nanoparticle-induced neurotoxicity.

## Planar lipid bilayer technique

Importantly, the neurotoxicity of particles depends not only on their size but also on their composition, shape, charge, surface properties, aggregation state, and ability to absorb toxic substances from the environment.^33^ Measurements of these characteristics require complex and sophisticated equipment, complicated techniques, and the existence of well-functioning biological models, for example cell culture, tissue preparations, and animals, which may not be readily amenable to direct testing in space flight. The complex characteristics of the particles determine their capacity to affect integrity of the plasma membrane of nerve terminals,^33,34^ which can be examined using corresponding membrane potential measurements. The capacity of particles to affect the parameters previously described could be assessed using a planar lipid bilayer, a well-known technique for investigation of ion-conducting properties of pore-forming molecules and proteins, ion channels, and transporters.^35–41^ Single, stable lipid bilayers between two compartments filled with saline were formed by Mueller et al.,^35^ and the transverse electrical properties were measured in controlled chemical investigations. Planar lipid bilayers are usually composed of phosphatidylcholine and cholesterol at a weight ratio of 2:1; however, other lipid and protein components can be also included.^36^ Bilayer membranes are usually painted across a round aperture with a small mm-sized diameter in a thin wall of a Teflon cup held within a glass chamber.^36^ A high-resolution amplifier and computer are used for voltage-clamp recordings of transmembrane current.^36^ Monitoring the membrane activity of micro-sized and nano-sized dust particles during long-term space flight could potentially be implemented using an adapted planar lipid bilayer technique through registration of artificial membrane conductivity in the presence of particles. Ion conductance of the planar lipid bilayer might correlate with the capacity of the acquired planetary dust particles, once added to the operation cuvette, to affect the membrane integrity. The planar lipid bilayer technique has already been proposed for investigation of the gravitational vector effects on action of antibiotic alamethicin.^37^

**Table 1:** Comparison of the ambient level of L-[^14^C]glutamate in the preparations of nerve terminals and their membrane potential registered as an increase in fluorescence (F = F_t_/F_0_) of the dye rhodamine 6 G in the presence of different types of micro- and nano-sized particles

Various types of micro- and nano-sized particles	The ambient level of L-[^14^C]glutamate (nmol/mg of protein) in the synaptosomal preparations	An increase in rhodamine 6 G fluorescence, (arb. units) in synaptosomes, reflecting membrane depolarization	References
Control synaptosomes without micro- and nano-sized particles	0.193 ± 0.013	0.015 ± 0.005	Krisanova et al. 2013;^22^ Pozdnyakova et al. 2017;^26^ Soika et al. 2017;^27^ Pozdnyakova et al. 2016;^25^ Borisova et al. 2017^24^
Martial soil simulant JSC, Mars-1A (2.0 mg/ml)	0.208 ± 0.021	0.017 ± 0.005	Krisanova et al. 2013;^22^ Pozdnyakova et al. 2017^26^
Lunar soil simulant JSC-1a (2.0 mg/ml)	0.214 ± 0.029	0.020 ± 0.005	Krisanova et al. 2013^22^
Nanocrystals NaYF_4_:Eu^3+^-PEG (0.5; 3.5; 7.5 mg/ml, respectively)	‒ ‒ 0.268 ± 0.017	0.02 ± 0.008; 0.05 ± 0.014; 0.11 ± 0.015	Soika et al. 2017^27^
Nanocrystals NaYF_4_:Eu-^3+^OH (0.5; 1.5; 3.5 mg/ml, respectively)	‒ ‒ 0.298 ± 0.012	0.04 ± 0.006; 0.06 ± 0.014; 0.14 ± 0.01	Soika et al. 2017^27^
Maghemite nanoparticles γ-Fe_2_O_3_ (0.75 mg/ml)	0.30 ± 0.02	0.10 ± 0.01	Horak et al. 2017;^28^ Borisova et al. 2014^29^
Nanodiamonds (1.0 mg/ml)	0.383 ± 0.022	0.10 ± 0.01	Pozdnyakova et al. 2016^25^
Carbon nanodots synthesized from thiourea (0.5 mg/ml; 1.0 mg/ml, respectively)	0.216 ± 0.01; 0.364 ± 0.02	0.07 ± 0.01 ‒	Borisova et al. 2017^24^

Initial feasibility of the planar lipid bilayer technique was shown in experiments with macrocycle calix[4]arene-bis-hydroxymethylphosphonic acid (calix[4]arene C-99) that demonstrated parallel calixarene-induced changes in the planar lipid membrane conductance and in the membrane potential of blood platelets.^36^ Polysaccharide-coated magnetite nanoparticles were inert regarding changes in the synaptosomal membrane potential and also demonstrated an absence of effects on the planar lipid membrane conductance.^38^ The vitamin B1 (thiamine) structural analogue 3-decyloxycarbonylmethyl-4-methyl-5-(β-hydroxyethyl) thiazole chloride changed the membrane potential of nerve terminals,^39^ and its sequential additions to the planar lipid bilayer membrane decreased the membrane stability and led to its eventual rupture at high concentrations.^40^ Simulated lunar and Martian dust particles did not affect the membrane potential of nerve terminals^22^ and also did not influence the conductance of the planar lipid bilayer.^41^ The principal similarity of effects obtained using neurochemical methods with those using the planar lipid bilayer technique was also found in preliminary experiments with carbon nanodots and nanodiamonds. Preliminary verification of the experimental data on particle neurotoxicity obtained using radiolabelled L-[^14^C]glutamate and [^3^H]GABA and fluorescence methods using fluorescent potential-sensitive dye with data on the membrane conductance measured using planar lipid bilayer equipment has been completed.^22–29,33,34,36,38,41^ However, extensive validation is required to have appropriate assessment algorithms and the parameter relations for different types of particles. Moreover, the lipid composition of the planar membrane can be easily varied (protein compounds might also be included to improve performance) and different parameters, e.g., membrane conductance and capacitance, can be analyzed.

It is difficult to make a predictive model for particle neurotoxicity without experimental measurements because of the huge diversity of particle characteristics and their unknown effects. Furthermore, even a small variation of particle size, charge, shape, and functional group exposure can unpredictably alter their neuroactive properties. In this context, the results from planar lipid bilayer measurements might supplement data obtained by other techniques. To support experimentation in space, the planar lipid bilayer technique would need to be adapted to be portable, automated, vibration-resistant, and suitable to perform measurements in a simple manner, thereby making the monitoring process manageable for those on board.

## Conclusions

With successful further optimization, it is possible that the planar lipid bilayer technique could be used for the rapid assessment of potential neurotoxicity of planetary and interstellar dust micro-sized and nano-sized particles during long-term space missions. If implemented, this approach might also be applicable to environmental monitoring for the assessment of toxicity of air pollution particulate matter on Earth and in nanotechnology for estimation of biosafety of engineered nanoparticles. Ultimately, it is hoped that the associated acquired knowledge could be of value for the prediction and prevention of harmful human health effects of particle inhalation.

### Reporting Summary

Further information on experimental design is available in the Nature Research Reporting Summary linked to this article.

## Supplementary information

Reporting Summary

## Supplementary information

**Supplementary information** accompanies the paper on the *npj Microgravity* website (10.1038/s41526-019-0062-7).

## References

[1] Gaier, J. R. The effects of lunar dust on EVA systems during the Apollo missions. *NASA/TM—2005-213610/REV*1 (2005).

[2] Latch JN, Hamilton RF, Holian A, James JT, Lam C (2008). Toxicity of lunar and Martian dust simulants to alveolar macrophages isolated from human volunteers. Inhal. Toxicol..

[3] Lam CW, James JT, Latch JNRFH, Holian A (2002). Pulmonary toxicity of simulated lunar and Martian dusts in mice: ii. biomarkers of acute responses after intratracheal instillation. Inhal. Toxicol..

[4] Chatterjee A, Wang A, Lera M, Bhattacharya S (2010). Lunar soil simulant uptake produces a concentration-dependent increase in inducible nitric oxide synthase expression in murine RAW 264.7 macrophage Cells. J. Toxicol. Environ. Health A.

[5] Lam C (2013). Toxicity of lunar dust assessed in inhalation-exposed rats. Inhal. Toxicol..

[6] Scully RR, Lam CW, James JT (2013). Estimating safe human exposure levels for lunar dust using benchmark dose modeling of data from inhalation studies in rats. Inhal. Toxicol..

[7] Air Quality Standards**/**Environment/European Commission (2018). http://ec.europa.eu/environment/air/quality/standards.htm.

[8] Block ML (2012). The outdoor air pollution and brain health workshop. Neurotoxicology.

[9] Cacciottolo M (2017). Particulate air pollutants, APOE alleles and their contributions to cognitive impairment in older women and to amyloidogenesis in experimental models. Transl. Psychiatry.

[10] Heusinkveld HJ (2016). Neurodegenerative and neurological disorders by small inhaled particles. Neurotoxicology.

[11] Casanova R (2016). A voxel-based morphometry study reveals local brain structural alterations associated with ambient fine particles in older women. Front. Hum. Neurosci..

[12] Kioumourtzoglou MA (2016). Long-term PM2.5 exposure and neurological hospital admissions in the Northeastern United States. Environ. Health Perspect..

[13] Maher BA (2016). Magnetite pollution nanoparticles in the human brain. Proc. Natl Acad. Sci. USA.

[14] Oberdörster G, Elder A, Rinderknecht A (2009). Nanoparticles and the brain: cause for concern?. J. Nanosci. Nanotechnol..

[15] Oberdörster G, Oberdörster E, Oberdörster J (2005). Nanotoxicology: an emerging discipline evolving from studies of ultrafine particles. Environ. Health Perspect..

[16] Wang J (2008). Potential neurological lesion after nasal instillation of TiO(2) nanoparticles in the anatase and rutile crystal phases. Toxicol. Lett..

[17] Takeda K (2009). Nanoparticles transferred from pregnant mice to their offspring can damage the genital and cranial nerve systems. J. Health Sci..

[18] Hapke BW, Cohen AJ, Cassidy WA, Wells EN (1970). Solar radiation effects in lunar samples. Science.

[19] Linnarsson D (2012). Toxicity of lunar dust. Planet. Space Sci..

[20] Loftus DJ, Rask JC, McCrossin CG, Tranfield EM (2010). The chemical reactivity of lunar dust: from toxicity to astrobiology physics and astronomy. Earth. Moon Planets.

[21] Costa-Fernández JM (2016). Mass spectrometry for the characterization and quantification of engineered inorganic nanoparticles. Trends Anal. Chem..

[22] Krisanova N (2013). Neurotoxic potential of lunar and Martian dust: influence on em, proton gradient, active transport, and binding of glutamate in rat brain nerve terminals. Astrobiology.

[23] Borisova T (2015). Neuromodulatory properties of fluorescent carbon dots: effect on exocytotic release, uptake and ambient level of glutamate and GABA in brain nerve terminals. Int. J. Biochem. Cell Biol..

[24] Borisova T (2017). Harmful impact on presynaptic glutamate and GABA transport by carbon dots synthesized from sulfur-containing carbohydrate precursor. Environ. Sci. Pollut. Res..

[25] Pozdnyakova N (2016). Neuroactivity of detonation nanodiamonds: Dose-dependent changes in transporter-mediated uptake and ambient level of excitatory/inhibitory neurotransmitters in brain nerve terminals. J. Nanobiotechnology.

[26] Pozdnyakova N (2017). Enrichment of inorganic Martian dust simulant with carbon component can provoke neurotoxicity. Microgravity Sci. Technol..

[27] Sojka B (2017). Effects of surface functionalization of hydrophilic NaYF4 nanocrystals doped with Eu(3+) on glutamate and GABA transport in brain synaptosomes. J. Nanopart. Res..

[28] Horák D (2017). Effect of O-methyl-β-cyclodextrin-modified magnetic nanoparticles on the uptake and extracellular level of L-glutamate in brain nerve terminals. Colloids Surf. B Biointerfaces.

[29] Borisova T (2014). Manipulation of isolated brain nerve terminals by an external magnetic field using D-mannose-coated γ-Fe_2_O_3_ nano-sized particles and assessment of their effects on glutamate transport. Beilstein J. Nanotechnol..

[30] Sudhof TC (2004). The synaptic vesicle cycle. Annu. Rev. Neurosci..

[31] Borisova T (2016). Permanent dynamic transporter-mediated turnover of glutamate across the plasma membrane of presynaptic nerve terminals: Arguments in favor and against. Rev. Neurosci..

[32] Borisova T, Borysov A (2016). Putative duality of presynaptic events. Rev. Neurosci..

[33] Borisova T (2018). Nervous system injury in response to contact with environmental, engineered and planetary micro- and nano-sized particles. Front. Physiol..

[34] Nixdorff K, Borisova T, Komisarenko S, Dando M (2018). Dual-use nano-neurotechnology: an assessment of the implications of trends in science and technology. Polit. Life Sci..

[35] Mueller P, Rudin DO, Tien HT, Wescott WC (1962). Reconstitution of cell membrane structure in vitro and its transformation into an excitable system. Nature.

[36] Shatursky OY (2014). Anion carrier formation by calix[4]arene-bis-hydroxymethylphosphonic acid in bilayer membranes. Org. Biomol. Chem..

[37] Hanke W (1996). Studies of the interaction of gravity with biological membranes using alamethicin doped planar lipid bilayers as a model system. Adv. Space Res..

[38] Borysov A (2014). A comparative study of neurotoxic potential of synthesized polysaccharidecoated and native ferritinbased magnetic nanoparticles. Croat. Med. J..

[39] Borisova, T. O. et al. The use of a compound of 3-decyloxycarbonylmethyl-4-methyl-5-(2-hydroxyethyl) thiazolium chloride as a compound that causes depolarization of the plasma membrane of isolated nerve terminals of the rat brain. Patent No. 45941 Ukraine, C07D277/00. Application No. u200909717 from 23.09.09; published 25.11.09, Bull. No. 22. National Medical University, Institute of Biochemistry, Institute of Biorganic Chemistry (2009).

[40] Shatursky O (2007). Vitamin B1 thiazole derivative reduces transmembrane current through ionic channels formed by toxins from black widow spider venom and sea anemone in planar phospholipid membranes. Biochim. Biophys. Acta.

[41] Borisova, T., Krisanova, N., Nazarova, A., Borysov, A., Chunihin, O. Lunar and Martian soil stimulants have different effects on L-[^14^C]glutamate binding to brain nerve terminals. Abstract id. F4.7-8-14. 40th COSPAR Scientific Assembly, 2–10 August. Moscow, Russia, (2014).

